# Extracellular Domains of CD8α and CD8ß Subunits Are Sufficient for HLA Class I Restricted Helper Functions of TCR-Engineered CD4^+^ T Cells

**DOI:** 10.1371/journal.pone.0065212

**Published:** 2013-05-30

**Authors:** Marleen M. van Loenen, Renate S. Hagedoorn, Renate de Boer, J. H. Frederik Falkenburg, Mirjam H. M. Heemskerk

**Affiliations:** Department of Hematology, Leiden University Medical Center, Leiden, The Netherlands; Institut Pasteur, France

## Abstract

By gene transfer of HLA-class I restricted T-cell receptors (TCRs) (HLA-I-TCR) into CD8^+^ as well as CD4^+^ T-cells, both effector T-cells as well as helper T-cells can be generated. Since most HLA-I-TCRs function best in the presence of the CD8 co-receptor, the CD8αß molecule has to be co-transferred into the CD4^+^ T-cells to engineer optimal helper T-cells. In this study, we set out to determine the minimal part of CD8αβ needed for optimal co-receptor function in HLA-I-TCR transduced CD4^+^ T-cells. For this purpose, we transduced human peripheral blood derived CD4^+^ T-cells with several HLA-class I restricted TCRs either with or without co-transfer of different CD8 subunits. We demonstrate that the co-transduced CD8αβ co-receptor in HLA-I-TCR transduced CD4^+^ T-cells behaves as an adhesion molecule, since for optimal antigen-specific HLA class I restricted CD4^+^ T-cell reactivity the extracellular domains of the CD8α and ß subunits are sufficient.

## Introduction

Adoptive transfer of T-cells is a strategy used to target both solid tumors[1] and leukemia[2]–[4]. By introducing well-characterised TCRs via gene transfer large numbers of T-cells with defined antigen-specificity can be obtained without long in vitro culture periods. Transfer of HLA-I-TCRs into CD8^+^ T-cells demonstrated redirected antigen-specificity[5]–[10] and recently the in vivo efficacy of adoptively transferred TCR transduced (td) T-cells was demonstrated in melanoma and synovial cell sarcoma patients[11]–[13]. For optimal maintenance of functional CD8^+^ immune responses in vivo, however, antigen-specific CD4^+^ T cells may play an essential role[14], [15]. By TCR engineering of CD8^+^ as well as CD4^+^ T-cells, both effector T-cells as well as helper T-cells with the same specificity can be generated. However, since most HLA-I-TCRs function best in the presence of the CD8 co-receptor, the CD8 molecule has to be co-transferred into the CD4^+^ T-cells to engineer optimal helper T-cells[16], [17].

The CD8 molecule can be expressed as an αα or an αß dimer, but is on peripheral TCRαß T-cells mostly expressed as an αß dimer[18]–[23]. The α subunit of CD8 binds to the non-polymorphic residues of the α3 domain of HLA class I, thereby enhancing the avidity of the TCR/MHC complex[24]. The cytoplasmatic tail of the α subunit directly associates with the protein tyrosine kinase Lck[25]–[28], promoting signal transduction after T-cell activation. The intracellullar domain of the ß subunit enhances the association of CD8α with lipid raft localized Lck[29], [30] and the linker for activation of T-cells (LAT)[31], [32]. Although the mechanism is not clear yet, it has been demonstrated that CD8αß heterodimers bind MHC class I molecules more avidly than CD8αα homodimers[32]–[34].

Previously, it was reported that for optimal proliferation, cytokine production and cytotoxicity of HLA-I-TCR td CD4^+^ T-cells co-expression of CD8αβ was needed whereas co-expression of CD8αα only marginally increased functional activity[16], [17]. Here, we studied whether the extracellular and/or intracellular part of CD8α and CD8ß were required for this increased functional activity.

## Results and Discussion

### Extracellular CD8α and ß are required and sufficient to elicit HLA class I restricted IFN-γ production

**Figure 1:**
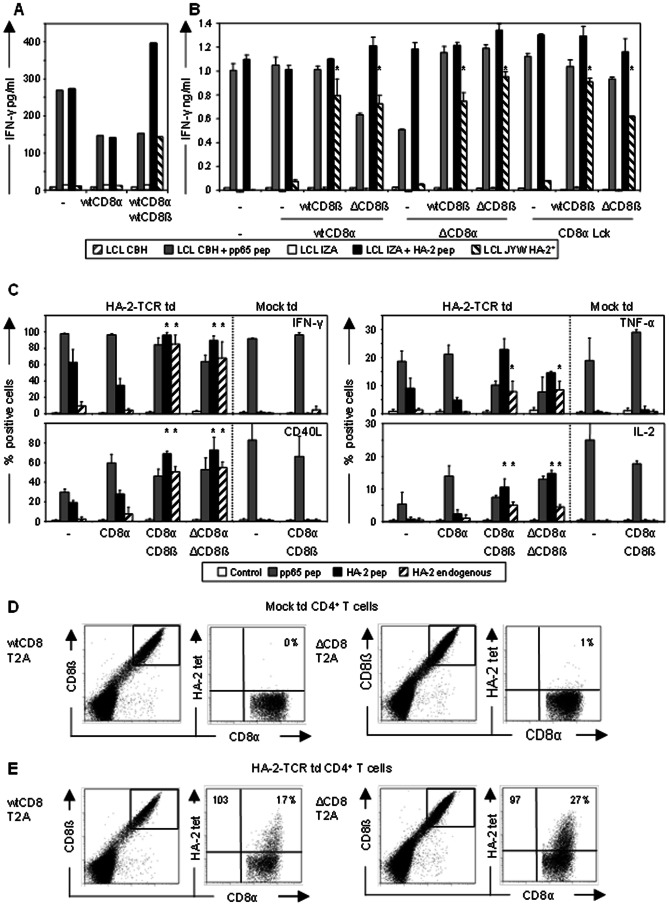
HLA-I-TCR td CD4^+^ T-cells co-transferred with wtCD8αß or intracellularly modified CD8αß demonstrate equal effector functions. To study the minimal part of CD8 needed for optimal co-receptor function in HLA-I-TCR td CD4^+^ T-cells, HA-2-TCR td CMV-specific CD4^+^ T-cells (A) co-transferred with wtCD8αα or wtCD8αß co-receptor, or (B) co-transferred with either wtCD8α,ΔCD8α or CD8α Lck in combination with either wtCD8ß or ΔCD8ß were purified and used in a stimulation assay. Td T-cell populations were tested against HLA-DR1^+^ LCL-CBH either unpulsed (grey striped bars) or pulsed with pp65 peptide (grey bars), or against HLA-A2^+^ HA-2^−^ LCL-IZA either unpulsed (white bars) or pulsed with HA-2 peptide (black bars), or against HLA-A2^+^ HA-2^+^ LCL-JYW (black striped bars). IFN-γ production was measured after 18 h of stimulation in duplicate, and a representative experiment out of 3 is depicted. The IFN-γ production of the different CD8αß expressing TCR td T-cells was compared to the IFN-γ production of CD8αα expressing TCR td T-cells within their group using students' t-test. P-values <0.05 are indicated with an asterisk. (C) To study whether co-transfer of CD8 would also result in polyfunctional helper functions of TCR td CMV-specific CD4^+^ T-cells, both mock and HA-2-TCR td CMV-specific CD4^+^ T-cells with or without co-transfer of different CD8 subunits as indicated in the figure were stimulated with HLA-DR1^+^ LCL-CBH pulsed with pp65 peptide (grey bars; pp65 pep), unpulsed HLA-A2^+^ HA-2^−^ LCL-IZA (white bars; control), HA-2 peptide pulsed HLA-A2^+^ HA-2^−^ LCL-IZA (black bars; HA-2 pep) or HLA-A2^+^ HA-2^+^ LCL-JYW (striped bars; HA-2 endogenous). After 5 h of stimulation, T-cells were stained with anti-IFN-γ, anti-TNF-α, anti-CD40L and anti-IL-2 mAbs and were analyzed using flow cytometry. The percentage of IFN-γ, TNF-α and IL-2 producing or CD40L expressing T-cells after stimulation is depicted. The percentages of cytokine producing and CD40L upregulating CD8αß expressing TCR td T-cells that were significantly higher than CD8 negative and CD8αα expressing TCR td T-cells (p-values <0.05) are indicated with an asterisk. (D/E) To study differences in avidity between HLA-I-TCR td CD4^+^ T-cells co-transferred with the different CD8α and CD8ß constructs, HA-2 tetramer staining was analyzed. (D) Mock or (E) HA-2-TCR td CD4^+^ T-cells co-transferred with either wtCD8α-T2A-wtCD8ß (wtCD8 T2A; left dot plots) or ΔCD8α-T2A-ΔCD8ß (ΔCD8 T2A, right dot plots) were stained with anti-CD8α and ß mAbs and HA-2-tetramers and analyzed using flow cytometry. Populations were gated on CD8αß positive expression and HA-2 tetramer staining is depicted for the gated populations. Percentages of HA-2-tetramer positive T-cells are indicated in the upper right and MFI of the HA-2-tetramer staining in the upper left of the dot plots. Data shown are representative for 2 independent experiments.

To verify that functional activity of high-affinity HLA-I-TCR transduced (td) CD4^+^ T-cells was improved by the transfer of CD8αα or CD8αß co-receptor, CMV-specific CD4^+^ T-cells were transduced with the high-affinity HA-2-TCR with either only CD8α or with both the CD8α and CD8ß subunits and purified based on CD8αα or CD8αß expression. T-cells were tested against LCLs pulsed with pp65 peptide stimulating the endogenous CMV-TCR, or with either HA-2 peptide or HA-2^+^ LCLs stimulating the introduced HA-2-TCR, and antigen-specific IFN-γ production was measured (Figure 1A). As can be observed, HA-2-TCR td CMV-specific CD4^+^ T-cells co-transferred with CD8αα, CD8αß or negative for CD8 were equally potent in recognizing pp65 peptide pulsed target cells. In addition, all three populations were able to recognize HA-2 peptide loaded target cells. However, only the CD8αß co-transferred T-cells were able to recognize endogenously processed and presented HA-2 (Figure 1A). These results confirmed previous studies demonstrating that retroviral introduction of CD8αβ increased the functional activity mediated via the introduced TCR of the TCR td CD4^+^ T-cells[16], [17].

To more precisely determine the part of the CD8αß coreceptor responsible for increased functional activity of the HLA-I-TCR td CD4^+^ T-cells, we constructed intracellularly truncated CD8α (ΔCD8α), Lck mutated CD8α (CD8α Lck), and intracellularly truncated CD8ß (ΔCD8ß). The HA-2-TCR td CMV-specific CD4^+^ T-cells were transduced with the different CD8α and CD8ß constructs, purified based on CD8αα or CD8αß expression, and used as effector T-cells in the experiments described here above (Figure 1B). Results were similar to the experiments with unmodified (wt)CD8α and wtCD8ß co-transferred HA-2-TCR td CMV-specific CD4^+^ T-cells (Figure 1A). Only TCR td CD4^+^ T-cells co-transferred with both CD8α and CD8β produced significant amounts of IFN-γ after stimulation with HA-2^+^ LCLs, irrespective of whether the co-transferred CD8α and ß subunits were intracellularly truncated or whether the Lck binding motif of the CD8α subunit was mutated (Figure 1B). Mock td CMV-specific CD4^+^ T cells specifically produced IFN-γ only after stimulation with pp65 peptide pulsed LCLs (data not shown). These results demonstrate that for optimal HLA class I restricted IFN-γ production of the TCR td CD4^+^ T-cells, co-transfer of the extracellular domains of CD8α and ß is required but that the intracellular domains can be dismissed.

Polyfunctionality of CD4^+^ T cells is important for optimal helper function. Therefore, we studied the capacity of HA-2-TCR td CMV-specific CD4^+^ T-cells co-transferred with different CD8 constructs to produce not only IFN-γ, but also TNF-α and IL-2 and upregulate CD40L (Figure 1C). TCR td CD4^+^ T-cells co-transferred with either wtCD8αß or ΔCD8αß produced significantly more cytokines and demonstrated significantly more CD40L upregulation after stimulation with either HA-2 pulsed or HA-2^+^ LCLs (p<0.05 indicated with asterisks) than CD8 negative or CD8αα expressing TCR td CD4^+^ T-cells. No significant difference in cytokine production or CD40L upregulation after HA-2 specific stimulation was observed between TCR td CD4^+^ T-cells co-transferred either with wtCD8αß or ΔCD8αß. The results in Figure 1C demonstrate that for IFN-γ, TNF-α and IL-2 production as well as for CD40L upregulation after stimulation with HA-2^+^ LCLs co-transfer of CD8α and ß, and most importantly the extracellular domains of these CD8 subunits, is required.

In conclusion, to generate polyfunctional HA-2-TCR td CMV-specific CD4^+^ T-cells, co-transfer of both CD8α and ß is required, but the intracellular domains of these CD8 subunits can be dismissed.

### ΔCD8α and ΔCD8ß improve HLA-class I restricted avidity similarly efficient as wtCD8α and wtCD8ß

To analyze whether TCR td CD4^+^ T-cells co-transferred with the different CD8 subunits bind with similar affinity the HLA-peptide complex, HA-2-tetramer staining was analyzed for both mock and HA-2-TCR td CD4^+^ T-cells (Figure 1D and E). No specific HA-2 tetramer staining could be observed for mock and TCR td CD4^+^ T-cells without CD8αß co-transfer (data not shown). However, co-transfer of CD8α alone or transfer of CD8αß using two separate retroviral vectors resulted in aspecific staining of every tetramer added (data not shown). Therefore, mock and HA-2-TCR td CMV-specific CD4^+^ T-cells were co-transferred with multicistronic vectors in which the CD8α and CD8ß molecules were linked with a 2A sequence resulting in equimolar levels of both CD8α and ß molecules (Figure 1D and E), and analyzed for tetramer staining. As can be seen in Figure 1D, no aspecific staining of HA-2 tetramer is detected on mock transduced CD4^+^ T-cells co-transferred with the multicistronic CD8αß vectors, whereas TCR td CD4^+^ T-cells co-transferred with the wtCD8αß or ΔCD8αß multicistronic vectors demonstrated identical HA-2 tetramer staining, indicating similar avidity for HLA-A2/HA-2-peptide complex (Figure 1E).

**Figure 2:**
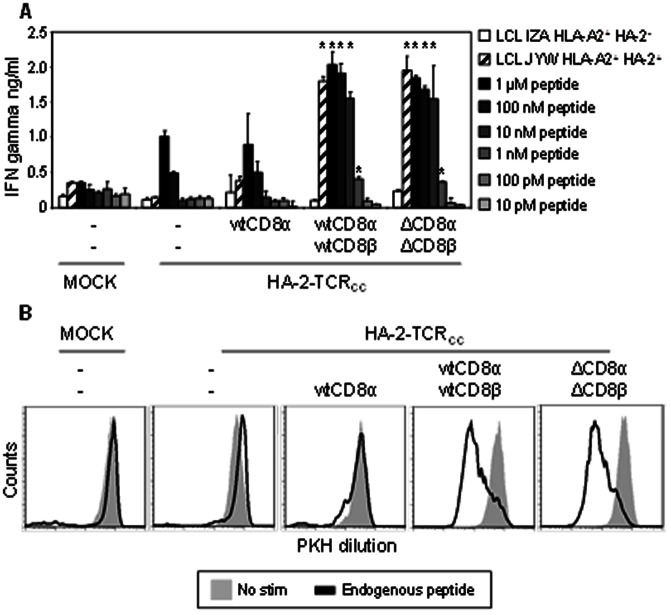
Improved HLA-class I restricted avidity of CD8αß expressing HA-2-TCR td CD4^+^ T-cells results in improved proliferation. (A) To study whether co-transfer of CD8 would also improve the peptide sensitivity of CD4^+^ T-cells transduced with a next generation HA-2-TCR, both mock and HA-2-TCR td CMV-specific CD4^+^ T-cells with or without co-transfer of different CD8 subunits as indicated in the figure were purified using flow cytometry based cell sorting and stimulated with unpulsed HLA-A2^+^ HA-2^−^ LCL IZA (white bars; LCL IZA), HLA-A2^+^ HA-2^−^ LCL-IZA pulsed with decreasing concentrations of HA-2 peptide (range 1 µM-10 pM) or HLA-A2^+^ HA-2^+^ LCL JYW (striped bars; LCL JYW). IFN-γ production was measured after 18 h of stimulation in duplicate, and a representative experiment out of 2 is depicted. The IFN-γ production of ΔCD8αß and wtCD8αß expressing HA-2-TCR_CC_ td CD4^+^ T-cells significantly higher (p-values <0.05) than CD8 negative or CD8αα expressing HA-2-TCR_CC_ td CD4^+^ T-cells is indicated with an asterisk. (B) To investigate their proliferative capacity, both mock and HA-2-TCR td CD4^+^ T-cells without CD8 or co-transferred with wtCD8α, wtCD8αß, or ΔCD8αß were purified based on markergene expression and CD8 cell surface expression and were either not stimulated (filled histograms) or stimulated with HLA-A2^+^ HA-2^+^ LCL-JYW (thick black line). Histograms depict PKH dilution measured 5 days after stimulation, and a representative example of 2 independent experiments is depicted.

Next, we studied whether co-transfer of wtCD8αß or ΔCD8αß equally improved the function of CD4^+^ T-cells transduced with a next generation HA-2-TCR_CC_, that was codon optimized and cysteine modified to improve TCR cell surface expression. For this purpose, CMV-specific CD4^+^ T-cells were transduced with the next generation HA-2-TCR_CC_ either without or in combination with different CD8 subunits, purified using flow cytometry based cell sorting and tested for HA-2-specific IFN-γ production against target cells loaded with titrated concentrations of the HA-2 peptide as well as against target cells that endogenously process and present the antigen (HA2^+^ target cells). As demonstrated in Figure 2A, the HA-2-TCR_CC_ td CD4^+^ T-cells expressing either wtCD8αß or ΔCD8αß were equally reactive against HA-2 peptide loaded target cells, and approximately a 100 fold more sensitive compared to CD8 negative or CD8αα expressing HA-2-TCR_CC_ td CD4^+^ T-cells. In addition, whereas CD8 negative or CD8αα expressing HA-2-TCR_CC_ td CD4^+^ T-cells demonstrated no or low reactivity against HA-2^+^ target cells, the ΔCD8αß and wtCD8αß expressing HA-2-TCR_CC_ td CD4^+^ T-cells were highly reactive against HA2^+^ target cells. To confirm the significantly increased sensitivity of the wtCD8αß and ΔCD8αß expressing HA-2-TCR_CC_ td CD4^+^ T-cells cells compared to the CD8 negative or CD8αα expressing cells, we performed a proliferation assay in which we stimulated PKH-labeled T-cells with HA-2^+^ and HA-2^−^ target cells and analyzed proliferation by measuring PKH dilution at day 5 after stimulation. As can be observed in Figure 2B, wtCD8αß and ΔCD8αß HA-2-TCR_CC_ td CD4^+^ T-cells proliferated equally efficient after stimulation with HA-2^+^ target cells, whereas for the CD8 negative or CD8αα expressing T-cells no antigen-specific proliferation was observed. These data indicate that co-transfer of the extracellular parts of CD8 increase avidity of HA-2-TCR expressing CD4^+^ T-cells for HLA-A2^+^ HA-2^+^ target cells approximately a 100-fold. This increase in avidity is necessary to elicit efficient IFN-γ production and proliferation of HA-2-TCR and HA-2-TCR_CC_ td CD4^+^ T-cells after stimulation with physiologically relevant levels of HA-2.

### In general, HLA-I-TCR td CD4^+^ T-cells require co-transfer of only the extracellular CD8αß domains

**Figure 3:**
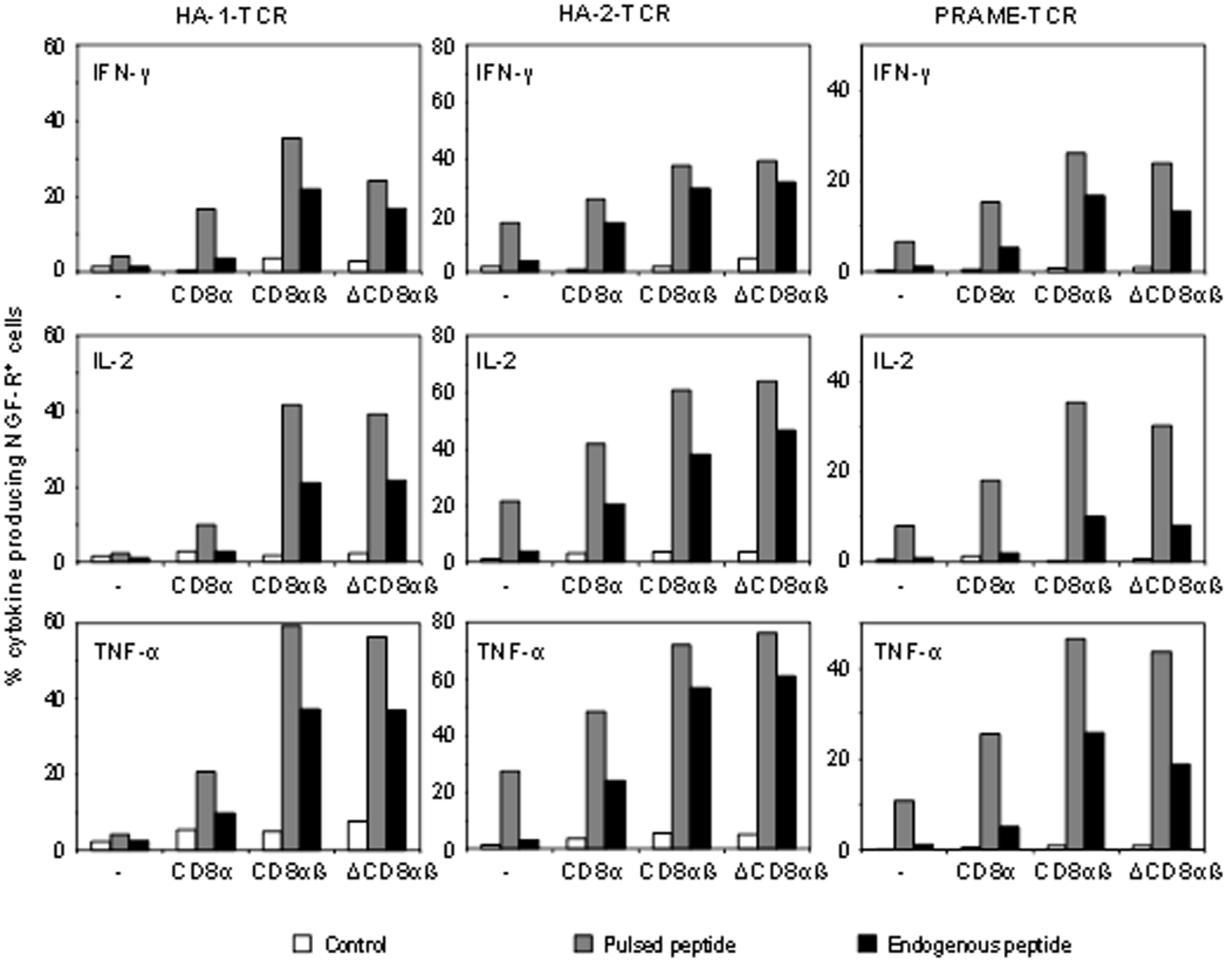
In general, co-transfer of the extracellular domains of CD8α and ß is required and sufficient. To confirm the generality of the previous data, total CD4^+^ T-cells were transduced with codon optimized and cysteine modified HA-1-, HA-2- or PRAME-TCR (transduction efficiency 48%, 48% and 22%, respectively) either with or without co-transfer of different CD8 molecules, as indicated in the figure. One week after transduction, non-purified TCR td CD4^+^ T-cells were stimulated and tested for cytokine production using flow cytometry. HA-1- or HA-2-TCR td CD4^+^ T-cells were stimulated either with HA-1 or HA-2 peptide pulsed or unpulsed HLA-A2^+^ HA-1^-^ HA-2^−^ LCL-IZA, or HLA-A2^+^ HA-1^+^ HA-2^+^ LCL-MRJ, and PRAME-TCR td CD4^+^ T-cells were stimulated either with PRAME peptide pulsed or unpulsed HLA-A2^+^ PRAME^−^ melanoma cells, or HLA-A2^+^ PRAME^+^ melanoma cells. 5 h After stimulation, T-cells were permeabilized and stained with anti-NGF-R in combination with either anti-IFN-γ (upper panel), anti-IL-2 (middle panel) or anti-TNF-α (lower panel), and analyzed using flow cytometry. The percentage of markergene positive and CD8 positive T-cells producing cytokines after stimulation with antigen-negative cells (white bars; control), peptide pulsed cells (grey bars; pulsed peptide) or antigen-positive cells (black bars; endogenous peptide) is indicated.

To confirm the generality of these data, polyclonal peripheral CD4^+^ T-cells were transduced with next generation high-affinity TCRs specific for HA-1, HA-2 or PRAME and were co-transferred with the different CD8 constructs. Results presented in Figure 3 demonstrate that CD4^+^ T-cells transduced with either HA-1-TCR_CC_, HA-2-TCR_CC_ or PRAME-TCR_CC_ displayed IFN-γ-, IL-2- and TNF-α production only after stimulation with peptide pulsed target cells (1 µg/ml), and not after stimulation with antigen-positive target cells expressing endogenously processed antigen. Introduction of only the wtCD8α molecule induced some cytokine production against peptide pulsed target cells and antigen-positive target cells. However, when wtCD8αß or ΔCD8αß were introduced, substantial percentages of transduced T-cells produced IFN-γ, IL-2 and TNF-α both after stimulation with peptide pulsed target cells or antigen-positive target cells. These results demonstrate a general trend in requirement and sufficiency of co-transfer of the extracellular domains of CD8α and ß for HLA-class I restricted helper functions.

## Conclusions

We hypothesize that CD8 co-transfer in antigen-experienced CD4^+^ T-cells potentially poses the risk of overstimulation. To minimize the risk of overstimulation in HLA class I restricted TCR transduced CD4^+^ T-cells, we analyzed in this study whether co-transfer of a signaling deficient CD8-co-receptor would also result in optimal HLA class I restricted functionality.

We confirmed that for optimal helper functions of HLA-I-TCR td CD4^+^ T-cells co-expression of CD8αß co-receptors is superior to CD8αα co-receptors, and CD8αß co-expressing T-cells were superior in producing IFN-γ, TNF-α, and IL-2, in upregulating CD40L, and in antigen specific proliferation. Expression of the extracellular domains of CD8αß was required both for CD4^+^ T-cells transduced with unmodified HA-2-TCR, as well as CD4^+^ T-cells transduced with codon optimized and cysteine modified TCRs. These modifications should result in higher cell surface expression due to improved translation and improved preferential pairing of the TCR-chains, but nevertheless CD8αß co-expression was required for robust HLA-class I restricted helper functions.

Introduction of the CD8αß co-receptor increased the sensitivity of the HLA-I-TCR td CD4^+^ T cells approximately a 100 fold, leading to efficient recognition of target cells that express the antigen endogenously. Truncation of the intracellular domains of the CD8α and CD8ß subunits did not change the functional properties of the HLA-I-TCR td CD4^+^ T-cells. Using CD4^+^ T-cells transduced with several different HLA-I-TCRs we confirmed the generality of the data.

Although it was demonstrated that both for CD8α[35] and CD8ß[36] the intra- as well as the extracellular domain play a role in positive selection of thymocytes, we demonstrate that for the effector function of peripheral T-cells the CD8αß co-receptor functions as an adhesive molecule rather than a signalling molecule. Therefore, to elicit robust helper functions in CD4^+^ T-cells transduced with high-affinity HLA class I restricted TCRs introduction of the extracellular domains of CD8α and ß subunits is required and sufficient. However, although we demonstrate equal proliferative capacity of HLA-I-TCR td CD4+ T-cells co-expressing either δCD8αß or wtCD8αß, it needs to be studied in vivo whether they also demonstrate equal long-term persistence in vivo.

## Materials and Methods

### Construction of retroviral vectors and production of retroviral supernatant

TCRα and TCRß chains of the HA-2-TCR[8], as well as of the codon optimized[37] and cysteine modified[38], [39] next generation HA-2-TCR_CC_, HA-1-TCR_CC_ [40], and PRAME-TCR_CC_ [41] were linked using a self-cleaving T2A sequence[42] and combined with the truncated nerve growth factor (NGF-R) into a retroviral vector. All TCRs used were CD8 dependent and HLA-A*0201-restricted. Both single vectors encoding unmodified CD8α (wtCD8α), truncated CD8α (ΔCD8α), Lck mutated CD8α (CD8α Lck), unmodified CD8ß (wtCD8ß) and truncated CD8ß (ΔCD8ß) as well as T2A linked wtCD8αß and ΔCD8αß constructs were engineered. ΔCD8α consists of amino acids (aa) 1-190, Lck mutated CD8α has mutations at position 201 and 203 (C→A), and ΔCD8ß consists of aa 1-176. CD8α constructs were combined with the eGFP markergene, whereas the CD8β constructs were combined with the NGF-R markergene. T2A linked wtCD8αß and ΔCD8αß constructs were engineered without a markergene. Retroviral vectors encoding eGFP or NGF-R alone were used as control vectors (mock). Using the Moloney murine leukemia virus-based retroviral vector LZRS and packaging cells φ-NX-A, viral supernatant was generated as previously described in detail[8].

### Flow cytometric analyses and cell sorting

For flow cytometric analyses as well as flow cytometry-based sorting, cells were labeled with tetramers for 1 h at 4°C or with mAbs directed against the various cell surface molecules for 30 minutes at 4°C. Cells were analyzed using the following mAbs: anti-CD3 APC-conjugated (Beckton Dickinson [BD], San Diego, CA, USA), anti-CD4 FITC-conjugated [BD], anti-NGF-R PE-conjugated [BD] or APC-conjugated (Cedarlane Laboratories, Hornby, Ontario, Canada), anti-BV2 PE-conjugated (Immunotech, Marseille, France) CD8α FITC- [BD], APC- [BD]or PE-conjugated (Invitrogen, Paisley, UK) and CD8ß PE-conjugated (Beckman Coulter, Fullerton, CA, USA). For cumulative measurement of several intracellular cytokines, the following mAbs were used: APC-labeled IFN-γ or IL-2 mAb or PE-labeled TNF-α or CD40L mAb [BD].

PE-labeled and APC-labeled HA-2 peptide-HLA tetramers were produced as described previously[43]. For data acquisition, a FACSCalibur was used and for data analysis FlowJo software was used.

### Donor CD4^+^ T-cells, stimulation and transduction

After study approval of the Leiden University Medical Center institutional review board and written informed consent according to the Declaration of Helsinki, peripheral blood mononuclear cells (PBMC) were obtained from hereditary hemochromatosis patients. From these PBMC samples, CMV-pp65-specific CD4^+^ T-cells were isolated as previously described[44]. Briefly^,^ PBMC were stimulated with 2 µg/ml pp65-KYQEFFWDANDIYRI (pp65 peptide; HLA-DRB1*0101-binding) peptide, and after 4 hours (h) of stimulation, IFN-γ secreting CD4^+^ T cells were isolated using the IFN-γ secretion assay (Miltenyi Biotec, Bergisch Gladbach, Germany). Subsequently, CMV-specific CD4^+^ T-cells were further purified by cell sorting based on TCR-BV2-staining. CMV-specific CD4^+^ T-cell line was stimulated with irradiated (30 Gy) allogeneic PBMCs (1×10^6^ cells/ml), and 800 ng/ml PHA (Murex Biotec Limited, Dartford, UK), and transduced 2 days after stimulation as described previously[45] with HA-2-TCR or empty vectors combined with NGF-R. After cell sorting based on NGF-R markergene expression, NGF-R markergene positive cells were re-stimulated, transduced with the different CD8 constructs, and sorted on basis of NGF-R expression, CD4 and CD8 cell surface expression. In addition, >94% MACS-enriched (Miltenyi Biotec) CD4^+^ T-cells derived from total PBMCs were transduced with either next generation HA-1-, HA-2-, or PRAME-TCR or empty vectors combined with NGF-R in combination with the different CD8 co-receptor subunits, and these T-cells were used in experiments without further purification.

### Analysis of cytokine production and proliferation

To test the capacity of T-cells to specifically proliferate in response to antigen, a PKH (St. Louis, Missouri, USA) based assay was used[46]. T-cells were labeled with PKH-26 (St. Louis, Missouri, USA) according to manufacturer's instructions, and 1×10^4^ T-cells were stimulated with 3×10^4^ target cells. PKH dilution was analyzed using flow cytometry at day 5 after stimulation.

To analyze reactivity of TCR td CMV-specific CD4^+^ T-cells, 5×10^3^ td T-cells were cocultured with 2×10^4^ target cells and after overnight incubation specific IFN-γ production was measured by standard ELISA[40]. For cumulative measurement of several intracellular cytokines, 1×10^5^ T-cells were stimulated with 2×10^5^ EBV-transformed lymphoblastoid cell lines (LCLs) in the presence of 10 µg/mL brefeldin A (BFA, Sigma-Aldrich, Zwijndrecht, The Netherlands), and 5 h after stimulation cytokine production was measured as previously described[44]. Targets used were HLA-typed LCL IZA (HLA-A*0201^+^ HA-2^−^), LCL JYW (HLA-A*0201^+^ HA-2^+^), LCL MRJ (HLA-A*0201^+^ HA-1^+^ HA-2^+^), or LCL CBH (HLA-DRB1*0101^+^) either unpulsed, or pulsed for 1 h at 37°C with 1 µg/ml HLA-DRB1*0101-binding pp65 peptide, or HLA-A2 binding HA-2 peptide (YIGVEVLVSV), or HA-1 peptide (VLHDDLLEA). In addition, HLA-A*0201^+^ PRAME^-^ and HLA-A*0201^+^ PRAME^+^ melanoma cells either unpulsed or pulsed with HLA-A*0201 binding PRAME peptide (SLLQHLIGL) were used as targets. All tests were performed in duplo.

### Statistics

Experimental data was evaluated in a paired fashion by use of the students' T-test. Reported *P* values are 2-sided and were considered statistically different if <0.05.

## References

[1] Dudley ME, Yang JC, Sherry R, Hughes MS, Royal R, et al (2008) Adoptive cell therapy for patients with metastatic melanoma: evaluation of intensive myeloablative chemoradiation preparative regimens. J Clin Oncol 26: 5233–5239. 18809613 10.1200/JCO.2008.16.5449 PMC2652090

[2] Collins RH Jr, Shpilberg O, Drobyski WR, Porter DL, Giralt S, et al (1997) Donor leukocyte infusions in 140 patients with relapsed malignancy after allogeneic bone marrow transplantation. J Clin Oncol 15: 433–444. 9053463 10.1200/JCO.1997.15.2.433

[3] Kolb HJ, Mittermuller J, Clemm C, Holler E, Ledderose G, et al (1990) Donor leukocyte transfusions for treatment of recurrent chronic myelogenous leukemia in marrow transplant patients. Blood 76: 2462–2465. 2265242

[4] Porter DL, Collins RH Jr, Hardy C, Kernan NA, Drobyski WR, et al (2000) Treatment of relapsed leukemia after unrelated donor marrow transplantation with unrelated donor leukocyte infusions. Blood 95: 1214–1221. 10666193

[5] Clay TM, Custer MC, Sachs J, Hwu P, Rosenberg SA, et al (1999) Efficient transfer of a tumor antigen-reactive TCR to human peripheral blood lymphocytes confers anti-tumor reactivity. J Immunol 163: 507–513. 10384155

[6] Cooper LJ, Kalos M, Lewinsohn DA, Riddell SR, Greenberg PD (2000) Transfer of specificity for human immunodeficiency virus type 1 into primary human T lymphocytes by introduction of T-cell receptor genes. J Virol 74: 8207–8212. 10933734 10.1128/jvi.74.17.8207-8212.2000 PMC112357

[7] Dembic Z, Haas W, Weiss S, McCubrey J, Kiefer H, et al (1986) Transfer of specificity by murine alpha and beta T-cell receptor genes. Nature 320: 232–238. 2421164 10.1038/320232a0

[8] Heemskerk MH, Hoogeboom M, de Paus RA, Kester MG, van der Hoorn MA, et al (2003) Redirection of antileukemic reactivity of peripheral T lymphocytes using gene transfer of minor histocompatibility antigen HA-2-specific T-cell receptor complexes expressing a conserved alpha joining region. Blood 102: 3530–3540. 12869497 10.1182/blood-2003-05-1524

[9] Heemskerk MH, Hoogeboom M, Hagedoorn R, Kester MG, Willemze R, et al (2004) Reprogramming of virus-specific T cells into leukemia-reactive T cells using T cell receptor gene transfer. J Exp Med 199: 885–894. 15051765 10.1084/jem.20031110 PMC2211874

[10] Kessels HW, Wolkers MC, van den Boom MD, van der Valk MA, Schumacher TN (2001) Immunotherapy through TCR gene transfer. Nat Immunol 2: 957–961. 11577349 10.1038/ni1001-957

[11] Johnson LA, Morgan RA, Dudley ME, Cassard L, Yang JC, et al (2009) Gene therapy with human and mouse T-cell receptors mediates cancer regression and targets normal tissues expressing cognate antigen. Blood 114: 535–546. 19451549 10.1182/blood-2009-03-211714 PMC2929689

[12] Morgan RA, Dudley ME, Wunderlich JR, Hughes MS, Yang JC, et al (2006) Cancer regression in patients after transfer of genetically engineered lymphocytes. Science 314: 126–129. 16946036 10.1126/science.1129003 PMC2267026

[13] Robbins PF, Morgan RA, Feldman SA, Yang JC, Sherry RM, et al (2011) Tumor regression in patients with metastatic synovial cell sarcoma and melanoma using genetically engineered lymphocytes reactive with NY-ESO-1. J Clin Oncol 29: 917–924. 21282551 10.1200/JCO.2010.32.2537 PMC3068063

[14] McKinney DM, Lewinsohn DA, Riddell SR, Greenberg PD, Mosier DE (1999) The antiviral activity of HIV-specific CD8+ CTL clones is limited by elimination due to encounter with HIV-infected targets. J Immunol 163: 861–867. 10395680

[15] Ahmed R, Gray D (1996) Immunological memory and protective immunity: understanding their relation. Science 272: 54–60. 8600537 10.1126/science.272.5258.54

[16] Kessels HW, Schepers K, van den Boom MD, Topham DJ, Schumacher TN (2006) Generation of T cell help through a MHC class I-restricted TCR. J Immunol 177: 976–982. 16818753 10.4049/jimmunol.177.2.976

[17] McNicol AM, Bendle G, Holler A, Matjeka T, Dalton E, et al (2007) CD8alpha/alpha homodimers fail to function as co-receptor for a CD8-dependent TCR. Eur J Immunol 37: 1634–1641. 17506031 10.1002/eji.200636900

[18] Baume DM, Caligiuri MA, Manley TJ, Daley JF, Ritz J (1990) Differential expression of CD8 alpha and CD8 beta associated with MHC-restricted and non-MHC-restricted cytolytic effector cells. Cell Immunol 131: 352–365. 2122925 10.1016/0008-8749(90)90260-x

[19] Goodman T, Lefrancois L (1988) Expression of the gamma-delta T-cell receptor on intestinal CD8+ intraepithelial lymphocytes. Nature 333: 855–858. 2968521 10.1038/333855a0

[20] Guy-Grand D, Cerf-Bensussan N, Malissen B, Malassis-Seris M, Briottet C, et al (1991) Two gut intraepithelial CD8+ lymphocyte populations with different T cell receptors: a role for the gut epithelium in T cell differentiation. J Exp Med 173: 471–481. 1824857 10.1084/jem.173.2.471 PMC2118788

[21] Janeway CA Jr (1992) The T cell receptor as a multicomponent signalling machine: CD4/CD8 coreceptors and CD45 in T cell activation. Annu Rev Immunol 10: 645–674. 1534242 10.1146/annurev.iy.10.040192.003241

[22] Rocha B, Vassalli P, Guy-Grand D (1991) The V beta repertoire of mouse gut homodimeric alpha CD8+ intraepithelial T cell receptor alpha/beta + lymphocytes reveals a major extrathymic pathway of T cell differentiation. J Exp Med 173: 483–486. 1824858 10.1084/jem.173.2.483 PMC2118783

[23] Zamoyska R (1994) The CD8 coreceptor revisited: one chain good, two chains better. Immunity 1: 243–246. 7889412 10.1016/1074-7613(94)90075-2

[24] Salter RD, Benjamin RJ, Wesley PK, Buxton SE, Garrett TP, et al (1990) A binding site for the T-cell co-receptor CD8 on the alpha 3 domain of HLA-A2. Nature 345: 41–46. 2109837 10.1038/345041a0

[25] Barber EK, Dasgupta JD, Schlossman SF, Trevillyan JM, Rudd CE (1989) The CD4 and CD8 antigens are coupled to a protein-tyrosine kinase (p56lck) that phosphorylates the CD3 complex. Proc Natl Acad Sci U S A 86: 3277–3281. 2470098 10.1073/pnas.86.9.3277 PMC287114

[26] Turner JM, Brodsky MH, Irving BA, Levin SD, Perlmutter RM, et al (1990) Interaction of the unique N-terminal region of tyrosine kinase p56lck with cytoplasmic domains of CD4 and CD8 is mediated by cysteine motifs. Cell 60: 755–765. 2107025 10.1016/0092-8674(90)90090-2

[27] Veillette A, Bookman MA, Horak EM, Bolen JB (1988) The CD4 and CD8 T cell surface antigens are associated with the internal membrane tyrosine-protein kinase p56lck. Cell 55: 301–308. 3262426 10.1016/0092-8674(88)90053-0

[28] Veillette A, Zuniga-Pflucker JC, Bolen JB, Kruisbeek AM (1989) Engagement of CD4 and CD8 expressed on immature thymocytes induces activation of intracellular tyrosine phosphorylation pathways. J Exp Med 170: 1671–1680. 2478653 10.1084/jem.170.5.1671 PMC2189493

[29] Arcaro A, Gregoire C, Bakker TR, Baldi L, Jordan M, et al (2001) CD8beta endows CD8 with efficient coreceptor function by coupling T cell receptor/CD3 to raft-associated CD8/p56(lck) complexes. J Exp Med 194: 1485–1495. 11714755 10.1084/jem.194.10.1485 PMC2193676

[30] Irie HY, Ravichandran KS, Burakoff SJ (1995) CD8 beta chain influences CD8 alpha chain-associated Lck kinase activity. J Exp Med 181: 1267–1273. 7699318 10.1084/jem.181.4.1267 PMC2191951

[31] Bosselut R, Zhang W, Ashe JM, Kopacz JL, Samelson LE, et al (1999) Association of the adaptor molecule LAT with CD4 and CD8 coreceptors identifies a new coreceptor function in T cell receptor signal transduction. J Exp Med 190: 1517–1526. 10562325 10.1084/jem.190.10.1517 PMC2195704

[32] Bosselut R, Kubo S, Guinter T, Kopacz JL, Altman JD, et al (2000) Role of CD8beta domains in CD8 coreceptor function: importance for MHC I binding, signaling, and positive selection of CD8+ T cells in the thymus. Immunity 12: 409–418. 10795739 10.1016/s1074-7613(00)80193-4

[33] Renard V, Romero P, Vivier E, Malissen B, Luescher IF (1996) CD8 beta increases CD8 coreceptor function and participation in TCR-ligand binding. J Exp Med 184: 2439–2444. 8976201 10.1084/jem.184.6.2439 PMC2196369

[34] Witte T, Spoerl R, Chang HC (1999) The CD8beta ectodomain contributes to the augmented coreceptor function of CD8alphabeta heterodimers relative to CD8alphaalpha homodimers. Cell Immunol 191: 90–96. 9973530 10.1006/cimm.1998.1412

[35] Fung-Leung WP, Louie MC, Limmer A, Ohashi PS, Ngo K, et al (1993) The lack of CD8 alpha cytoplasmic domain resulted in a dramatic decrease in efficiency in thymic maturation but only a moderate reduction in cytotoxic function of CD8+ T lymphocytes. Eur J Immunol 23: 2834–2840. 8223860 10.1002/eji.1830231117

[36] Itano A, Cado D, Chan FK, Robey E (1994) A role for the cytoplasmic tail of the beta chain of CD8 in thymic selection. Immunity 1: 287–290. 7889416 10.1016/1074-7613(94)90080-9

[37] Scholten KB, Kramer D, Kueter EW, Graf M, Schoedl T, et al (2006) Codon modification of T cell receptors allows enhanced functional expression in transgenic human T cells. Clin Immunol 119: 135–145. 16458072 10.1016/j.clim.2005.12.009

[38] Boulter JM, Glick M, Todorov PT, Baston E, Sami M, et al (2003) Stable, soluble T-cell receptor molecules for crystallization and therapeutics. Protein Eng 16: 707–711. 14560057 10.1093/protein/gzg087

[39] Kuball J, Dossett ML, Wolfl M, Ho WY, Voss RH, et al (2007) Facilitating matched pairing and expression of TCR chains introduced into human T cells. Blood 109: 2331–2338. 17082316 10.1182/blood-2006-05-023069 PMC1852191

[40] van Loenen MM, de Boer R, Hagedoorn RS, van Egmond EH, Falkenburg JH, et al (2011) Optimization of the HA-1-specific T-cell receptor for gene therapy of hematologic malignancies. Haematologica 96: 477–481. 21109688 10.3324/haematol.2010.025916 PMC3046283

[41] Amir AL, van der Steen DM, van Loenen MM, Hagedoorn RS, de Boer R, et al (2011) PRAME-specific Allo-HLA-restricted T cells with potent antitumor reactivity useful for therapeutic T-cell receptor gene transfer. Clin Cancer Res 17: 5615–5625. 21771875 10.1158/1078-0432.CCR-11-1066

[42] Szymczak AL, Workman CJ, Wang Y, Vignali KM, Dilioglou S, et al (2004) Correction of multi-gene deficiency in vivo using a single 'self-cleaving' 2A peptide-based retroviral vector. Nat Biotechnol 22: 589–594. 15064769 10.1038/nbt957

[43] Burrows SR, Kienzle N, Winterhalter A, Bharadwaj M, Altman JD, et al (2000) Peptide-MHC class I tetrameric complexes display exquisite ligand specificity. J Immunol 165: 6229–6234. 11086057 10.4049/jimmunol.165.11.6229

[44] Zandvliet ML, van LE, Jedema I, Kruithof S, Kester MG, et al (2011) Simultaneous isolation of CD8(+) and CD4(+) T cells specific for multiple viruses for broad antiviral immune reconstitution after allogeneic stem cell transplantation. J Immunother 34: 307–319. 21389867 10.1097/CJI.0b013e318213cb90

[45] Heemskerk MH, Hagedoorn RS, van der Hoorn MA, van der Veken LT, Hoogeboom M, et al (2007) Efficiency of T-cell receptor expression in dual-specific T cells is controlled by the intrinsic qualities of the TCR chains within the TCR-CD3 complex. Blood 109: 235–243. 16968899 10.1182/blood-2006-03-013318

[46] van Loenen MM, Hagedoorn RS, Kester MG, Hoogeboom M, Willemze R, et al (2009) Kinetic preservation of dual specificity of coprogrammed minor histocompatibility antigen-reactive virus-specific T cells. Cancer Res 69: 2034–2041. 19223543 10.1158/0008-5472.CAN-08-2523

